# Optimal combinations of fluorescent vessel labeling and tissue clearing methods for three-dimensional visualization of vasculature

**DOI:** 10.1117/1.NPh.9.4.045008

**Published:** 2022-11-30

**Authors:** Jingtan Zhu, Yating Deng, Tingting Yu, Xiaomei Liu, Dongyu Li, Dan Zhu

**Affiliations:** a Britton Chance Center for Biomedical Photonics–MoE Key Laboratory for Biomedical Photonics, Wuhan National Laboratory for Optoelectronics–Advanced Biomedical Imaging Facility, Huazhong University of Science and Technology, Wuhan, Hubei, China; b Optics Valley Laboratory, Wuhan, Hubei, China

**Keywords:** tissue optical clearing, vessel labeling, optical imaging, intact organs

## Abstract

**Significance:**

Visualization of intact vasculatures is crucial to understanding the pathogeneses of different neurological and vascular diseases. Although various fluorescent vessel labeling methods have been used in combination with tissue clearing for three-dimensional (3D) visualization of different vascular networks, little has been done to quantify the labeling effect of each vessel labeling routine, as well as their applicability alongside various clearing protocols, making it difficult to select an optimal combination for finely constructing different vasculatures. Therefore, it is necessary to systematically assess the overall performance of these common vessel labeling methods combined with different tissue-clearing protocols.

**Aim:**

A comprehensive evaluation of the labeling quality of various vessel labeling routines in different organs, as well as their applicability alongside various clearing protocols, were performed to find the optimal combinations for 3D reconstruction of vascular networks with high quality.

**Approach:**

Four commonly-used vessel labeling techniques and six typical tissue optical clearing approaches were selected as candidates for the systematic evaluation.

**Results:**

The vessel labeling efficiency, vessel labeling patterns, and compatibility of each vessel labeling method with different tissue-clearing protocols were quantitatively evaluated and compared. Based on the comprehensive evaluation results, the optimal combinations were selected for 3D reconstructions of vascular networks in several organs, including mouse brain, liver, and kidney.

**Conclusions:**

This study provides valuable insight on selecting the proper pipelines for 3D visualization of vascular networks, which may facilitate understanding of the underlying mechanisms of various neurovascular diseases.

## 1 Introduction

The cardiovascular system is a complex network containing numerous blood vessels, from large arteries/veins to small capillaries. This extensive vascular system plays an extremely essential role in maintaining normal physiology by supplying oxygen and nutrients, as well as eliminating metabolic wastes and supporting restoration after acute injuries or diseases.^1^^–^^3^ Therefore, visualizing three-dimensional (3D) vascular networks down to the capillary level helps to understand the underlying mechanisms of many vessel-associated diseases and design effective clinical therapies. In recent years, studies examining vascular architectures and their adaptive changes in various biological tissues under pathological conditions have been numerous.^4^^,^^5^ Traditional medical imaging techniques, such as computed tomography (CT), magnetic resonance imaging (MRI), and positron emission tomography (PET), can capture macrovessels within whole organs; however, their limited resolutions render the effective identification of a single capillary impossible.

Modern optical imaging methods can provide excellent resolutions for imaging complex vessel networks down to the capillary level.^6^^,^^7^ However, nearly all tissue types possess high scattering characteristics, which severely restrict the imaging depth of optical tomography. In the past several years, emerging tissue optical clearing techniques have used physical and chemical approaches to successfully overcome light scattering and absorption within tissues, hence improving imaging depth and quality.^8^^,^^9^ Combining vessel labeling, tissue clearing, and optical imaging practices provides an important tool for accurate mapping of vasculature in different tissue types, as well as vascular remodeling assessments after the occurrence of acute injuries or vessel-associated diseases.^10^^,^^11^

The effective labeling of vascular networks with bright fluorescence is the first and an important step for 3D imaging of vascular structures. So far, a variety of fluorescent vessel labeling methods have been employed to label vascular structures; these can be roughly divided into three categories based on labeling principles: genetically encoded fluorescent protein labeling, vessel-specific marker labeling, and fluorescent dye filling labeling.^4^ Of these, vasculature labeling with vessel-specific markers and fluorescent dye filling are the two most popular strategies in tissue clearing research on whole vasculatures, as demonstrated by the multiple studies conducted.^12^^–^^15^ For example, vessel-specific markers, such as lectins, are used in combination with 3DISCO to study vascular details in the brain and spinal cord.^16^ Lectins are also used alongside ScaleS or CUBIC clearing methods to visualize intact vascular structures.^17^^,^^18^ Additionally, the filling of lipophilic dyes or gelatin-based solutions is employed for vessel labeling in m-xylylenediamine (MXDA)-based clearing system (MACS) and CLARITY clearing methods, respectively.^19^^,^^20^ However, given the diverse demands for labeling and clearing different tissues using different approaches, few investigations have systematically quantified the labeling efficiency of each vessel labeling technique in different organs, as well as their applicability with various clearing protocols. Hence, selecting the best-fit pipeline for respective experiments remains a massive challenge to researchers because of the lack of sufficient quantitative data. Therefore, systematic evaluations of the labeling efficiencies of common vessel labeling methods, as well as their compatibilities with major tissue clearing procedures, must be comprehensively carried out.

In this study, we chose four commonly used vessel-labeling approaches from two labeling strategies: vessel-specific marker injection (lectin and CD31 antibodies) and fluorescent dye filling (Gel-Dex-FITC and DiI), and we quantitatively assessed their vessel labeling efficiencies in different mouse organs. We also analyzed the vessel distribution patterns of the four labeling procedures in mouse brains and other typical organs. Next, we chose six well-established tissue clearing techniques: uDISCO,^21^ FDISCO,^22^ PEGASOS,^23^ CUBIC,^24^ MACS,^19^ and PACT,^25^ and we comprehensively tested and quantified their compatibilities with the above vessel labeling methods. Finally, based on the robust comparative results, we screened out selected vessel labeling methods combined with the clearing protocols for the 3D reconstruction of vascular networks in typical mouse organs. The established optimal vessel labeling/tissue clearing combinations allow for 3D visualization and analysis of acute vascular lesions in a traumatic brain injury (TBI) mouse model. This research will provide valuable information to researchers looking to choose best-fit pipelines to visualize entire vascular networks in histological and pathological applications.

## 2 Methods

### 2.1 Animals

Wild-type mice (C57BL/6J, 8 to 12 weeks old) were used in this study. The animals were housed in a specific-pathogen-free (SPF) animal house under a 12/12 h light/dark cycle with unrestricted access to food and water. All animal experiments were performed under the Experimental Animal Management Ordinance of Hubei Province, P. R. China, and the Huazhong University of Science and Technology guidelines and approved by the Institutional Animal Ethics Committee of Huazhong University of Science and Technology.

### 2.2 TBI Mouse Models

The mice were anesthetized with 4% isoflurane in a $N_{2}O/O_{2}$ (70%/30%) mixture and maintained with 1.5% isoflurane in the same mixture for the whole surgery. The scalps of the mice were cut to expose the skull surface. The body temperature was maintained at 37°C±0.5°C with a heating pad. The injury was triggered via a controlled cortical impact device consisting of a fixed impactor using the following parameters: a tube diameter of 4 mm, impact speed of $3\;m\;s^{-1}$, and impact depth of 1 mm. The resulting injury was obvious with these parameters. The mice then went through the vascular labeling procedure as follows before having the chance to wake up.

### 2.3 Sample Preparation

Vascular labeling using Gel-Dex-FITC. We used the protocol described by Tsai et al. with slight modifications.^26^ A gelatin solution was prepared by dissolving porcine skin gelatin (no. G1890; Sigma-Aldrich) in hot 0.01 M phosphate-buffered saline (PBS, Sigma, P3813). FITC-conjugated dextran powder was dissolved in a 2% (w/v) gelatin solution at a concentration of 0.5% (w/v), and the mixed solution was kept at 40°C to 45°C before use. For vascular labeling, anesthetized mice were transcardially perfused first with a 0.01 M PBS solution and then 10 mL of fluorescent solutions. Perfused mice bodies were then transferred to a low-temperature environment (e.g., 4°C) for rapid cooling and solidification of the gelatin solution in vessels. After cooling, the desired mouse organs were extracted and fixed in 4% paraformaldehyde (PFA, Sigma-Aldrich, 158127) overnight at 4°C.

Vascular labeling with DiI. The protocol used for DiI labeling was described previously.^27^ DiI powder (Aladdin, D131225) was dissolved in 100% ethanol at a concentration of 6  mg/mL to prepare a stock solution, which can be stored in the dark at room temperature for up to 1 year. A DiI working solution was prepared by adding 200  μL of the DiI stock solution to 10 mL of diluent (0.01 M PBS and 5% (wt/vol) glucose at a ratio of 1:4), as reported previously.^27^ A DiI working solution should be freshly made before use. Anesthetized mice were first perfused with 0.01 M PBS at a rate of 1 to 2  mL/min (total 3 to 4 min) and then with 10 to 15 mL of the DiI working solution at a rate of 1 to 2  mL/min (total 10 to 15 min); the color of the mice’s ears, noses, and palms should become slightly purple during the perfusion of the DiI solution. Finally, desired organs were harvested and postfixed in 4% PFA overnight.

Vascular labeling with lectin and CD31 antibodies. The protocol used for lectin and CD31 antibodies was described previously.^15^^,^^22^ A DyLight 649 conjugated L. esculentum (Tomato) lectin (LEL-D649, DL-1178, Vector Laboratories) and an Alexa Fluor 647 conjugated anti-mouse CD31 antibody (CD31-A647, 102416, BioLegend) were also used to label the vasculature. Lectin was diluted in saline to a concentration of 0.5  mg/mL and injected into the tail vein (0.1 mL per mouse). The Alexa Fluor 647 conjugated anti-mouse CD31 antibody (10 to 15 mg) was diluted in saline and injected into the tail vein (total volume of 200  μL per mouse). Anesthetized mice were then transcardially perfused with 0.01 M PBS. The desired organs (e.g., brains, livers, kidneys) were excised from the perfused animal bodies. All harvested samples were postfixed in 4% PFA overnight at 4°C.

The fixed tissues were sliced using a commercial vibratome (Leica VT 1200 s, Germany).

### 2.4 Tissue Clearing Protocols

We chose different types of tissue clearing methods: hydrophobic clearing procedures, including FDISCO, uDISCO, and PEGASOS; hydrophilic clearing techniques, including CUBIC and MACS; and a hydrogel-based protocol, PACT. All evaluated clearing protocols were performed according to the instructions in the original publications. The clearing procedures for each method are briefly summarized as follows.

FDISCO.^22^ The FDISCO procedure consists of two steps: dehydration and RI matching. First, the fixed tissues are dehydrated using tetrahydrofuran (THF) (Sinopharm Chemical Reagent Co., Ltd., Shanghai, China) solutions at concentration gradients of 50%, 70%, 80%, and 100%. The pH of each THF solution is adjusted to 9.0 by adding triethylamine. The dehydrated tissues are then incubated in pure dibenzyl ether (DBE) (Aladdin, Shanghai, China) for RI matching. The time for each dehydration step (50% to 100% THF) is 4 h (tissue slices)/12 h (whole organs), and the time for RI matching with DBE is 4 h (tissue slices)/12 h (whole organs). All steps are conducted at 4°C, with gentle shaking.

uDISCO.^21^ Dehydration reagents are prepared by mixing pure tert-butanol (Sigma-Aldrich, St. Louis) with distilled water at upgraded concentrations of 30%, 50%, 70%, 80%, 90%, 96%, and 100% (v/v). The fixed tissues are sequentially dehydrated in each reagent. The dehydrated tissues are then immersed in dichloromethane and BABB-D4 until the samples become transparent. The time for each dehydration step (30% to 100% tert-butanol) is 4 h (tissue slices)/12 h (whole organs), and the time for RI matching with BABB-D4 is 4 h (tissue slices)/12 h (whole organs). The dehydration steps are performed at 35°C, and the RI matching steps are performed at room temperature, with gentle shaking.

PEGASOS.^23^ The samples are first decolorized with a 25% Quadrol decolorization solution. The samples are then dehydrated in *tert*-Butanol solution gradients, tB-PEG (70% v/v tert-Butanol, 27% v/v PEG methacrylate Mn 500 (PEGMMA500) (Sigma-Aldrich, 409529), and 3% w/v Quadrol (Sigma-Aldrich, 122262)). Next, the samples are immersed in BB-PEG [75% v/v benzyl benzoate (BB) (SigmaAldrich B6630) and 25% v/v PEGMMA500 (Sigma-Aldrich, 409529) containing a 3% w/v Quadrol (Sigma-Aldrich, 122262) added] medium for clearing. The time for decolorization is 12 h, and the time for each dehydration step and final RI matching is 4 h. All steps are performed at 37°C, with gentle shaking.

CUBIC.^24^ CUBIC-L is prepared by mixing 10% (wt/wt) N-butyldiethanolamine (Tokyo Chemical Industry, B0725) with 10% (wt/wt) Triton X-100 (Sigma-Aldrich, T8787) in ${\mathrm{dH}}_{2}O$. CUBIC-R is prepared by mixing 45% (wt/wt) antipyrine (Tokyo Chemical Industry, D1876) with 30% (wt/wt) nicotinamide (Tokyo Chemical Industry, N0078) in ${\mathrm{dH}}_{2}O$. The samples are incubated in CUBIC-L for delipidation for 1.5 d, washed with PBS for 6 h, and then exposed to CUBIC-R for RI matching for 0.5 d. All steps are performed at 37°C, with gentle shaking.

MACS.^19^ The fixed samples are serially incubated in MACS-R0, MACS-R1, and MACS-R2 solutions at room temperature while shaking gently. MACS-R0 is prepared by mixing 20% (vol/vol) MXDA (Tokyo chemical industry, D0127), 15% (wt/vol) sorbitol (Sigma, 85529), and ${\mathrm{dH}}_{2}O$; MACS-R1 is prepared by mixing 40% (vol/vol) MXDA with 30% (wt/vol) sorbitol dissolved in 1× PBS. MACS-R2 is prepared by mixing 40% (vol/vol) MXDA with 50% (wt/vol) sorbitol in ${\mathrm{dH}}_{2}O$. The incubation time for MACS-R0, MACS-R1, and MACS-R2 is 6, 2, and 1 h (tissue slices)/36, 12, and 12 h (whole organs), respectively. All steps are performed at 30°C, with gentle shaking.

PACT.^25^ The PACT clearing method is comprised of three steps: hydrogel embedding, delipidation, and RI matching. First, the samples are incubated in A4P0 hydrogel solution (4% acrylamide in 0.01 M PBS), doused with 0.25% photoinitiator 2,20-Azobis [2-(2-imidazolin-2-yl) propane] dihydrochloride (Wako Chemicals, Osaka, Japan) at 4°C overnight, and incubated at 37°C for 6 h to achieve polymerization. Next, the embedded samples are washed with 0.01 M PBS and subjected to delipidation in 8% sodium dodecyl sulfate (SDS) (Sinopharm Chemical Reagent Co., Ltd., Shanghai, China) in 0.01 M PBS. The samples are again washed with PBS and immersed in a sorbitol-based refractive index matching solution (sRIMS) (75% (wt/vol) sorbitol solution) until the tissues become transparent. The time for delipidation with SDS is 96 h, and the time for RI matching is 24 h. All steps are performed at 37°C, with gentle shaking.

### 2.5 Imaging

Fluorescent images of the cleared samples were captured using a light sheet microscope (LiToneXL, Light Innovation Technology, China) equipped with a 4× objective lens [NA=0.28, working distance (WD)=20  mm]. Thin light sheets were illuminated from all four sides of the sample, and a merged image was obtained. For light sheet imaging, the laser power was set to 10%, and the exposure time was 100 to 200 ms. An inverted laser-scanning confocal fluorescence microscope (LSM710, Zeiss, Germany) was used for the fluorescence imaging of tissue sections, employing the 5× objective lens (FLUAR, NA=0.25, WD=12.5  mm) and 10× objective lens (FLUAR, NA=0.5, WD=2  mm). Data were collected using the Zen 2011 SP2 (Version 8.0.0.273, Carl Zeiss GmbH, Germany) software. For confocal imaging, the laser power of 488 and 561 channels was set to 5%, and the laser power of 633 channels was set to 15% because the total power of 633 lasers is only a third of that of the 488/561 lasers. The gains were set to 500 to 600 to make sure that the image was not overexposure. The scan speed was set to 8. The image pre- and post-clearing for each labeling method was imaged under the same conditions.

### 2.6 Image Data Processing

All raw image data were collected in a lossless TIFF format (8-bit images for confocal microscopy and 16-bit images for light-sheet microscopy). The 16-bit images were transformed into 8-bit images by ImageJ to enable fast processing by other software, such as Imaris. Processing and 3D rendering were executed on a Dell workstation with an 8-core Xeon processor, 256 GB RAM, and an Nvidia Quadro P2000 graphics card. 3D and 2D image visualizations were conducted using Imaris (Version 7.6, Bitplane AG) and ImageJ (Version 1.51n), respectively. Tile scans from light-sheet microscopy were stitched utilizing MATLAB (Version 2014a, Mathworks).

### 2.7 Quantifications

To evaluate the efficiency of the different vessel labeling methods, as well as the fluorescence compatibility of each vessel labeling technique with different clearing procedures, signal-to-background ratios were calculated. First, the image stacks (50-μm-thickness) were imported in ImageJ. The “threshold” function in ImageJ was used to extract the vascular signals for each image in an image stack. Then, the “plot Z-axis profile” function in ImageJ was applied to calculate the mean signal intensities for each image. Next, three areas without blood vessels were manually selected as background regions for each image in the image stack. The mean values of these areas were determined as the background intensities of each image. The signal-to-background ratios for each image in the stack were defined as the mean signal intensities divided by the mean background intensities. The final signal-to-background ratio for an image stack was determined as the mean value of signal-to-background ratios for each image in the stack. Additionally, given the background heterogeneity in different brain regions, we calculated the signal-to-background ratio for three different brain regions and used the mean value as the final signal-to-background ratio for each labeling method to reduce the influence caused by the background heterogeneity.

3D vascular reconstruction and quantification by Imaris was performed using the pipeline described in previous studies. In brief, the “Threshold (loops)” algorithm in the filaments module was used to trace and quantify blood vessels. The parameter for the filament diameter was automatically set by the algorithm according to the voxel size. The voxel size of our data is 0.83  μm×0.83  μm×2  μm. The threshold for extracting vascular information was manually adjust to ensure the complete recognition of all blood vessels. After reconstruction of the vascular network, the local vessel density, average radius, and vessel segment length were obtained using the “statistics-.selection” in the filaments module.

### 2.8 Statistical Analysis

**Fig. 1:**
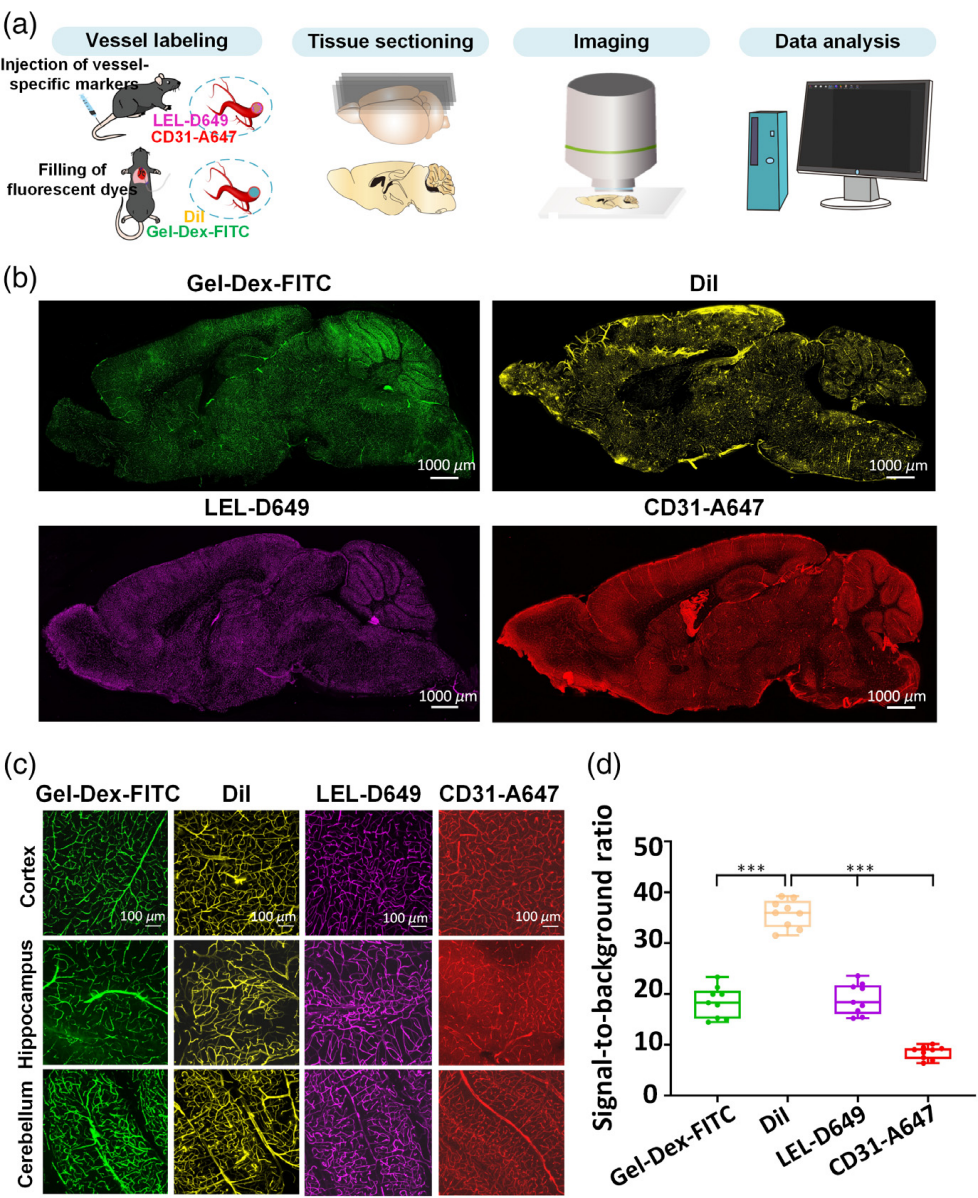
Comparison of the labeling efficiency of four selected vessel labeling methods in the mouse brain. (a) Experimental workflow for vessel labeling, imaging, and analysis. (b) Fluorescence images of overall vessel information in sagittal mouse brain slices labeled with Gel-Dex-FITC, DiI, LEL-D649, and CD31-A647 antibodies, respectively. (c) Fluorescence images of vessel information in three typical brain regions at high magnification, including the cortex, hippocampus, and cerebellum. (d) Signal-to-noise ratios of labeled vasculatures using different methods (n=6). All values are presented as the mean±SD. Statistical significance in (d) (***, P<0.001) was assessed with one-way ANOVA, followed by the Bonferroni post hoc test.

**Fig. 2:**
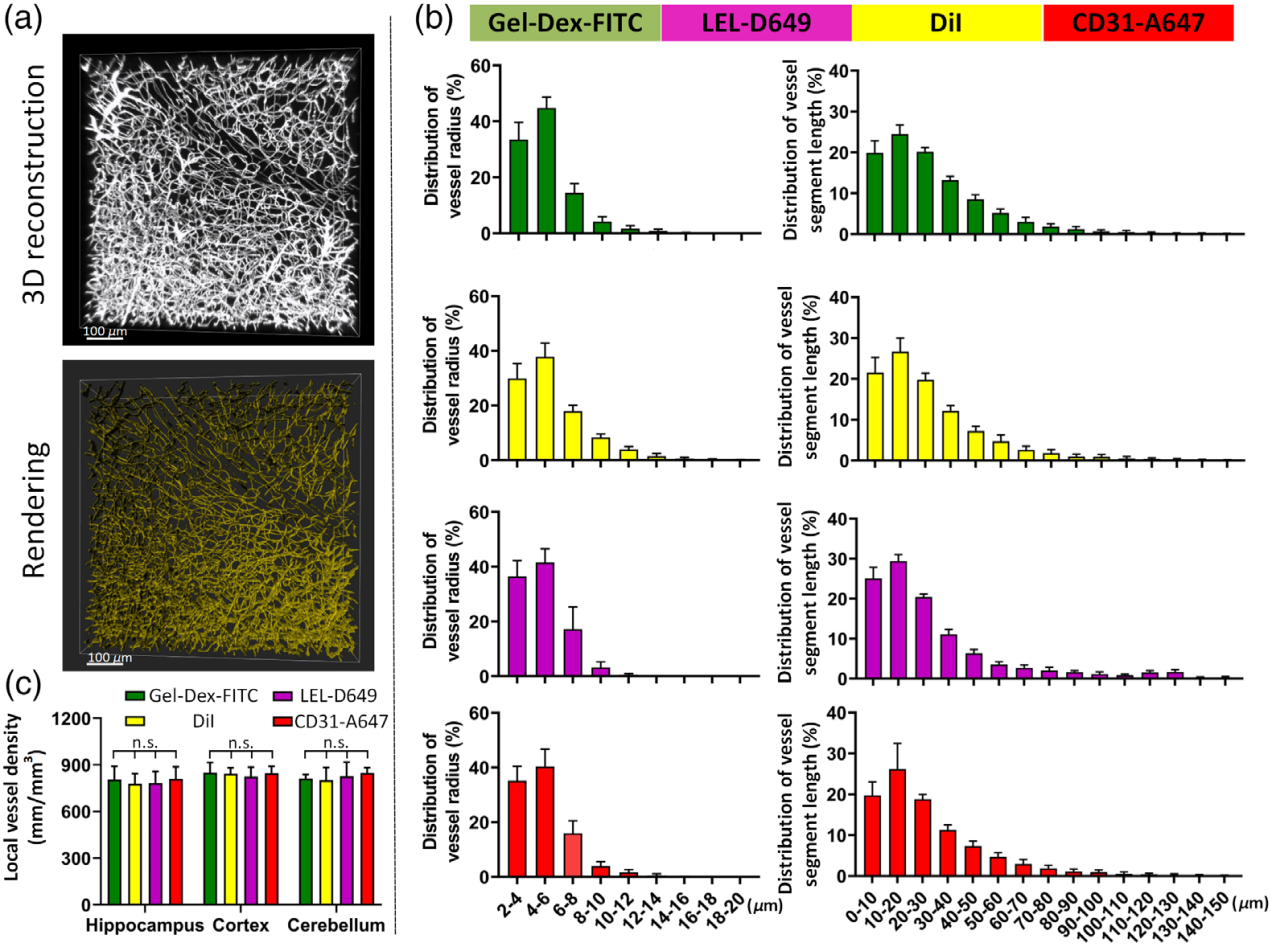
Analysis of vascular networks in the brain labeled using four vessel-labeling methods. (a) Representative images of the reconstruction and analysis of brain vessels. (b) Statistical analyses of the distributions of vessel radii and lengths of individual vessel segments, respectively (n=6). (c) Quantification of local vessel densities of specific brain regions in the brain, including the hippocampus, cortex, and cerebellum (n=6). All values are presented as the mean ± SD. Statistical significance in (c) (n.s., P>0.05) was assessed using one-way ANOVA, followed by the Bonferroni post hoc test.

Data are presented as the mean ± SD and were analyzed using the SPSS software (Version 22, IBM, USA), with 95% confidence intervals. The sample sizes are indicated in the figure legends. For the analysis of statistical significance, the normality of the data distribution in each experiment was checked using the Shapiro–Wilk test. The variance homogeneity for each group was evaluated employing Levene’s test. P values were calculated using an independent sample t-test (two-sided) to compare data between two groups, as shown in Figs. 4(a)–4(d) and Figs. 6(e)–6(f). P values were determined using one-way ANOVA, followed by the Bonferroni post hoc test to compare data, as shown in Figs. 1(d) and 2(c). P<0.05 was considered significant (*, P<0.05; **, P<0.01; ***, P<0.001).

## 3 Results

### 3.1 Comparing the Labeling Efficiency of the Four Selected Vessel Labeling Methods

Efficiently labeling entire vasculatures is fundamental to understanding the organization and structure of vascular networks. Generally, there are two types of widely employed fluorescent vascular labeling strategies in tissue clearing-related research for obtaining vascular information: vessel-specific marker vessel labeling and fluorescent dye filling vessel labeling. Vessel-specific markers, such as CD31 antibodies and lectins, are often directly injected into live mice through the tail vein for sufficient circulation to the entire vascular system. Another common strategy for labeling vessels is to fill the vessel lumen with fluorescent dyes, such as fluorescence-conjugated dextran mixed with gelatin and lipophilic dyes. Based on a comprehensive literature review, we selected four commonly used vessel labeling methods: the injection of two widely-used vessel-specific markers [DyLight 649 conjugated L. esculentum (Tomato) lectin (LEL-D649)], an Alexa Fluor 647 conjugated anti-CD31 antibody (CD31-A647), and common fluorescent dye filling (Gel-Dex-FITC and DiI).

Vascular structures in mouse brains were labeled using the four aforementioned methods based on protocols described in the literature.^15^^,^^19^^,^^22^^,^^26^ The labeled mouse brains were sagittally sliced into sections and imaged with a confocal microscope. The entire pipeline is shown in Fig. 1(a). Figure 1(b) displays fluorescence images of the vascular structures labeled with Gel-Dex-FITC, DiI, LEL-D649, and CD31-A647 antibodies, respectively. All of the assessed techniques revealed similar and uniform labeling effects on the brain vasculature, both in the entire collection of sagittal slices and in three typical brain regions, including the cortex, hippocampus, and cerebellum, at high magnification [Figs. 1(b) and 1(c)].

We calculated the signal-to-noise ratios of the fluorescence images from samples labeled using different approaches to evaluate the vessel labeling efficiency of each method. As shown in Fig. 1(d), the signal-to-noise ratio of the lipophilic dye DiI-obtained vasculature was higher than those of vasculatures stained using other protocols. The signal-to-noise ratio of the intravenous injection of LEL-D649-labeled vasculature labeled by intravenous injection of LEL-D649 was similar to the vasculature labeled by transcardial perfusion by Gel-Dex-FITC. Vasculature labeled with the intravenous injection of the CD31-A647 antibody demonstrated a relatively low signal-to-noise ratio compared with other evaluated labeling procedures.

### 3.2 Evaluating Vessel Distribution Patterns Created by Different Labeling Methods

Vascular networks in different organs often span several scales from micron-sized capillaries to large vessels extending over several millimeters.^12^ It can sometimes be challenging to achieve the complete labeling of entire vasculatures in various organs using a single approach. Therefore, evaluating the distribution patterns of vessels in different organs labeled using different methods is necessary.

We first assessed the labeling patterns created by the four labeling techniques on microvessels in mouse brain tissues by calculating the distribution of the vessel radius and vessel segment length, which are usually used to evaluate the vessel distribution pattern [Fig. 2(a)]. As shown in Fig. 2(b), vascular structures labeled using the four methods exhibited a similar distribution pattern both in vascular diameter and vessel segment length, which are similar to the results in recent publications using perfusion-based vessel labeling and the same quantification procedures,^28^^,^^29^ namely the diameters of over 95% of the labeled vessels were <12  μm, and about 70% of vessel segment length were shorter than 50  μm. These results indicate that microvessels accounted for the majority of the entire vascular composition in the brain. Additionally, we also calculated the vessel density in three typical brain regions labeled by the four methods: the cortex, hippocampus, and cerebellum [Fig. 2(c)]. The density of labeled vascular networks is similar among the four labeling methods in the three brain regions, consistent with the quantitative results displayed in previous studies.^10^^,^^28^ In general, all four examined labeling techniques are capable of realizing the fine labeling of brain microvessels in a similar pattern.

**Fig. 3:**
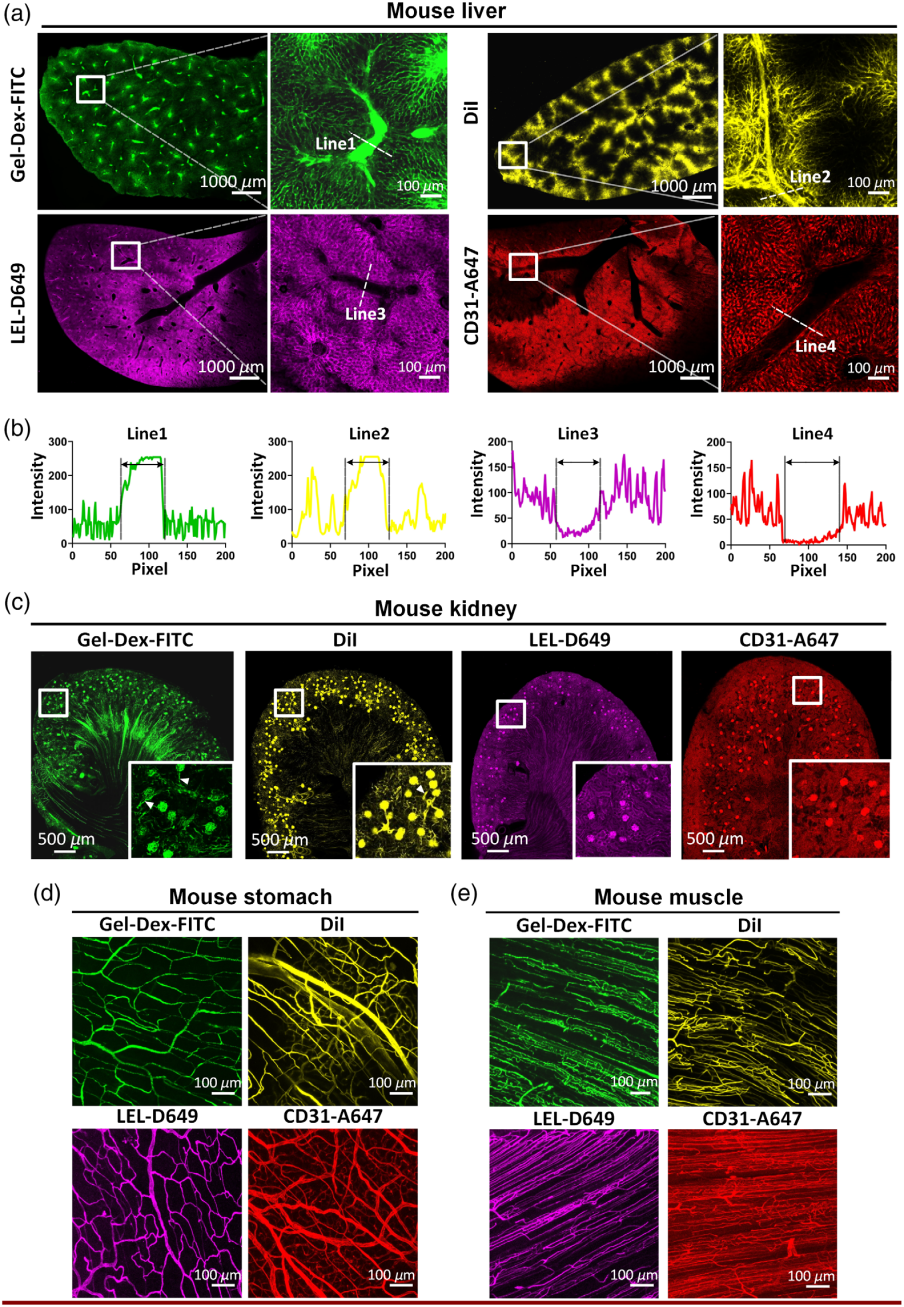
Comparison of the labeling efficiency of four selected vessel labeling methods in typical mouse organs. (a) Fluorescence images of overall vessel information and magnification of boxed regions of mouse liver slices labeled using Gel-Dex-FITC, DiI, LEL-D649, and CD31-A647, respectively. (b) Signal profiles of large vessels marked in the magnified images in (a). (e) Fluorescence images of the overall vasculature and magnification of boxed regions of mouse kidney slices labeled using the four methods. (d) Fluorescence images of labeled vessels in the mouse stomach characterized using the four methods. (e) Fluorescence images of labeled vessels in the mouse muscle characterized using the four methods.

In addition to the brain, we also labeled and imaged vascular networks in different mouse organs. For mouse liver, the capillary networks could be efficiently labeled by all of the tested four methods; however, the uniformity of labeled vasculature by perfusion with DiI is obviously weaker than the other three methods, probably due to the potential dye leakage [Fig. 3(a)]. Additionally, we discovered that large blood vessels were not marked by intravenous injection of CD31-A647 antibody and LEL-D649, leaving unlabeled cavities within vessel lumens [Fig. 3(b)]. In contrast, large vessels could be effectively casted by transcardial perfusion with Gel-Dex-FITC or DiI [Fig. 3(b)]. As for mouse kidney, we also found that vasculature labeled intravenously via the injection of the CD31-A647 antibody and LEL-D649 showed only the glomeruli information, with most of the information for the surrounding vessels being lost [Fig. 3(c)]. For other microvessel-dominated organs, such as the stomach and muscles, the four labeling techniques all achieved better labeling performance [Figs. 3(d) and 3(e)]. Based on these results, we speculated that vessel labeling using intravenous injections of specific markers efficiently labeled microvessels but not some large vessels, whereas vessel labeling via perfusion of dye solution tended to label both large vessels and capillaries.

### 3.3 Assessing the Fluorescence Compatibility of Vessel Labeling with Current Tissue Clearing Methods

The compatibility of the vessel labeling with current tissue-clearing techniques is another important factor in realizing the fine reconstruction of entire vascular networks in different organs. Here, we selected six typical tissue clearing methods: solvent-based methods, such as uDISCO,^21^ FDISCO,^22^ and PEGASOS,^23^ and aqueous-based approaches, such as CUBIC,^24^ PACT,^25^ and MACS.^19^ We examined the compatibility of each clearing procedure with vessel labeling using the four vessel-labeling methods.

**Fig. 4:**
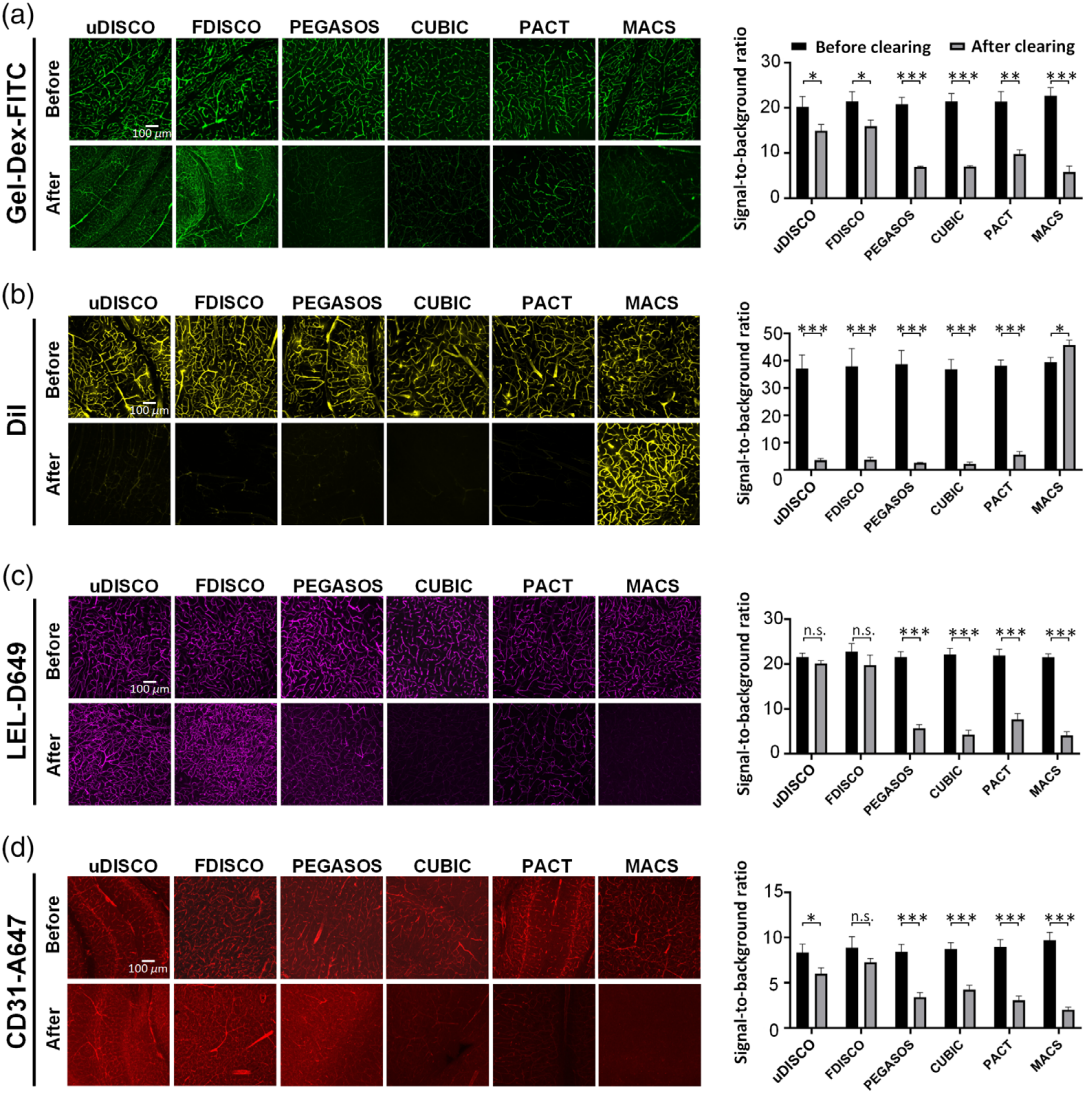
Evaluation of the fluorescence compatibilities of four vessel-labeling methods with tissue clearing. (a) Fluorescence images and quantification of signal-to-background ratios in mouse brain slices labeled using Gel-Dex-FITC before and after clearing with each clearing method. (b) Fluorescence images and quantification of signal-to-background ratios in mouse brain slices labeled using DiI before and after clearing with each clearing method. (c) Fluorescence images and quantification of signal-to-background ratios of mouse brain slices labeled using LEL-D649 before and after clearing with each clearing method. (d) Fluorescence images and quantification of signal-to-background ratios of mouse brain slices labeled using CD31-A647 before and after clearing with each clearing method. All values are presented as the mean ± SD. Statistical significance in (a) to (d) (***, P<0.001; **, P<0.01; *, P<0.05; n.s., P>0.05) was assessed using an independent-sample t-test.

Labeled vascular information in brain samples was imaged before and after clearing with each tissue-clearing method. Signal-to-background ratios pre- and post-clearing were also calculated to quantitatively assess fluorescence preserving capabilities. As shown, all assessed clearing methods decreased the signal-to-background ratio of vessel information labeled using Gel-Dex-FITC perfusion to different degrees after clearing, with uDISCO and FDISCO performing better than the other procedures [Fig. 4(a)]. For vessel information labeled using DiI solution perfusion, the compatibility of most of the clearing methods with DiI was weak; only the MACS clearing approach preserved the fluorescence signals well [Fig. 4(b)]. As for vasculature labeled through intravenous injections of LEL-D649 and CD31-A647 antibodies, the uDISCO and FDISCO methods showed excellent protections for the labeled signals, resulting in no obvious loss of signal-to-background ratios; by contrast, other evaluated clearing methods, especially for aqueous based clearing methods, noticeably decreased the signal-to-background ratios to different degrees after clearing [Figs. 4(c) and 4(d)]. These results suggest that each vessel labeling technique has preferable applicability for certain types of tissue clearing. For instance, Gel-Dex-FITC, LEL-D649, and CD31-A647 antibodies are preferable for solvent-based clearing methods (except PEGASOS, which uses Quadrol solution), such as uDISCO and FDISCO, and DiI is more suitable for detergent- and solvent-free clearing methods, such as MACS.

These quantitative comparison results for evaluating the labeling performance for each labeling method, as well as the compatibilities of each labeling method with different clearing methods, are summarized in Table 1.

**Table 1:** Comparison of the overall performance of different vessel labeling methods.

	Labeling methods	Labeling quality^a^	Labeling integrality		Optical clearing compatibility						Organ types recommended for the optical combinations	Tissue processing time^c^
			Large vessel	Microvessel	uDISCO	FDISCO	PEGASOS	CUBIC	PACT	MACS		
Tail vein injection-based	LEL-D649	+++	++	++++	++++^b^	++++^b^	+	+	+	+	Brains, stomach, muscles, or other microvessel-oriented organs	2 to 5 days, depending on tissue size
	CD31-A647	++	++	++++	+++	++++^b^	++	+	+	+	Brains, stomach, muscles, or other microvessel-oriented organs	2 to 5 days, depending on tissue size
Perfusion-based	GEL-DEX-FITC	+++	+++	++++	+++^b^	+++^b^	+	+	++	+	Liver, kidney, or other organs with considerable large vessels	2 to 5 days, depending on tissue size
	DiI	++++	++++	++++	+	+	+	+	+	++++^b^	Liver, kidney, or other organs with considerable large vessels	1 to 4 days, depending on tissue size

a Based on the signal-to-noise ratio data for each labeling method.

b Indicates the optimal tissue optical clearing methods for each vessel labeling method.

c The entire tissue processing time for the optimal combinations varied from tissue sections to whole organs.

### 3.4 3D visualization of Vascular Networks in Typical Organs for Health and Disease

Combining vessel labeling with tissue clearing techniques enables the visualization of the 3D morphology details of vasculature using optical imaging procedures, therefore, allowing researchers to examine pathological changes in overall vessel networks during the occurrences of different diseases. Here, based on results from comprehensive evaluations, we selected the optimal combination of fluorescent vessel labeling and tissue-clearing methods to construct vascular networks for several typical organs.

**Fig. 5:**
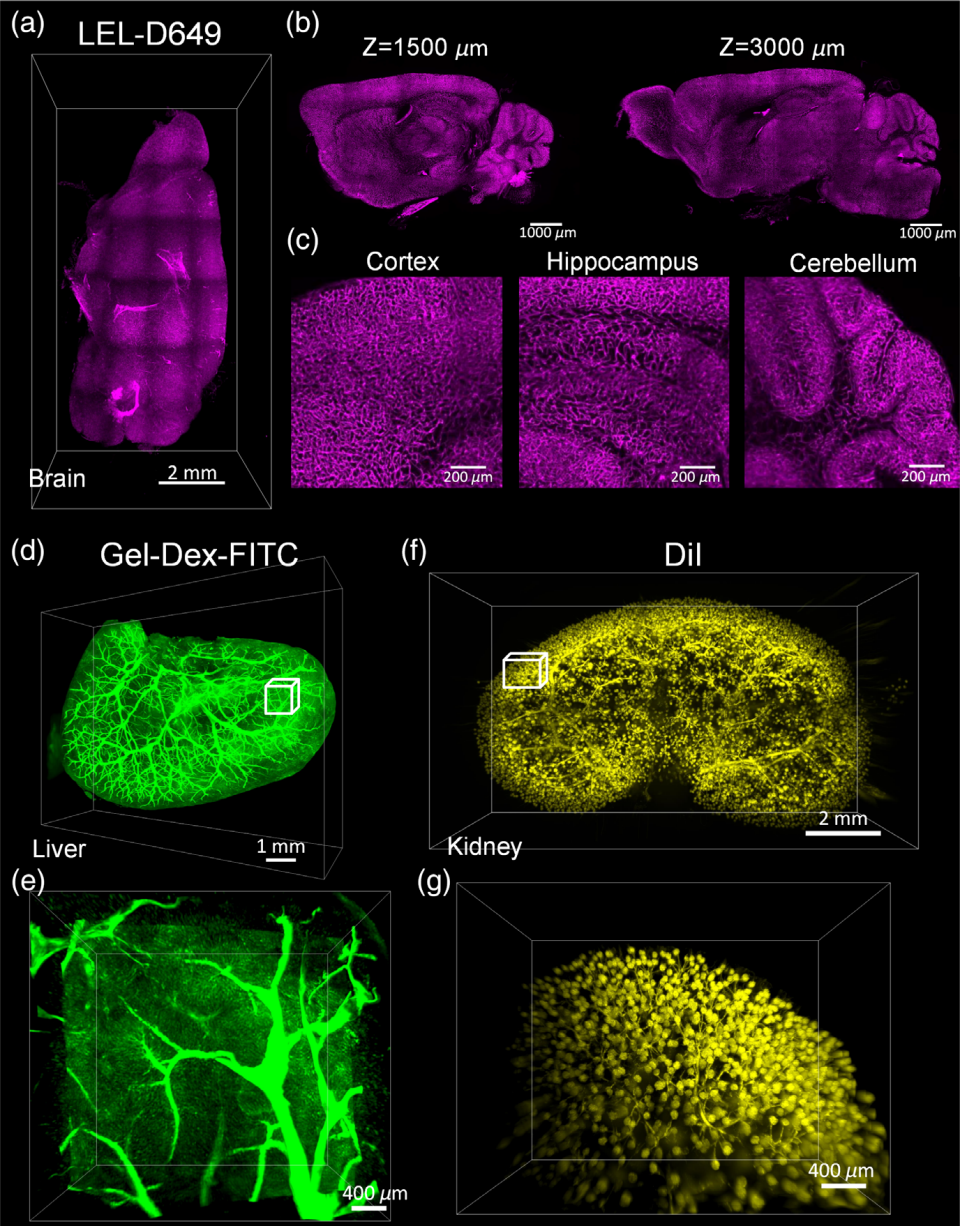
3D reconstruction of vascular networks in typical organs using the selected combination of vessel labeling and clearing methods. (a) 3D reconstruction of microvascular architecture in the mouse brain hemisphere labeled using LEL-D649 and cleared using uDISCO (Video 1, .mp4, 28.8 MB [URL: https://doi.org/10.1117/1.NPh.9.4.045008.s1]). (b) Acquired optical section images at different imaging depths. (c) High-magnification images of vascular structures in different brain regions. (d) 3D rendering of vascular networks in the mouse liver labeled using Gel-Dex-FITC and cleared using FDISCO (Video 2, .mp4, 29.5 MB [URL: https://doi.org/10.1117/1.NPh.9.4.045008.s2]), (e) Magnification of the boxed regions in (d). (f) 3D reconstruction of glomerular tufts and vessels in the mouse kidney labeled using DiI and cleared using MACS. (g) Magnification of the boxed regions in (f) (Video 3, .mp4, 29.6 MB [URL: https://doi.org/10.1117/1.NPh.9.4.045008.s3]).

The mouse brain hemisphere labeled using LEL-D649 was cleared by uDISCO to obtain imaging datasets for 3D reconstruction [Fig. 5(a), Video 1]. The overall vascular information at different depths was acquired, and microvessel structures were well observed in different brain regions, including the cortex, hippocampus, and cerebellum [Figs. 5(b) and 5(c)]. As for the mouse liver, the Gel-Dex-FITC solution perfusion procedure was chosen for vascular labeling and FDISCO for clearing; the 3D reconstruction of liver vasculature is shown in Fig. 5(d) and Video 2. Both the large vessels and capillaries were well defined [Fig. 5(e)]. Finally, for clearing DiI-labeled mouse kidneys, the MACS method was selected because it is the only clearing approach capable of preserving the DiI-labeled fluorescent signals. As shown in Figs. 5(f) and 5(g) and Video 3, both the glomerulus trees and branches in kidneys were clearly visible in DiI-labeled MACS-cleared kidneys.

In addition to healthy tissues, 3D visualization and quantification of microvascular remodeling under certain pathological conditions are also essential for biomedical studies. For example, TBI is a kind of serious brain injury induced by an external force and is a major cause of death and disability for human beings. TBI will cause severe neurovascular injury to brains, leading to chronic global neurological impairments.^30^^,^^31^ Here, we used the selected optimal vessel labeling/tissue clearing combinations to investigate the cerebrovascular damage from TBI.

**Fig. 6:**
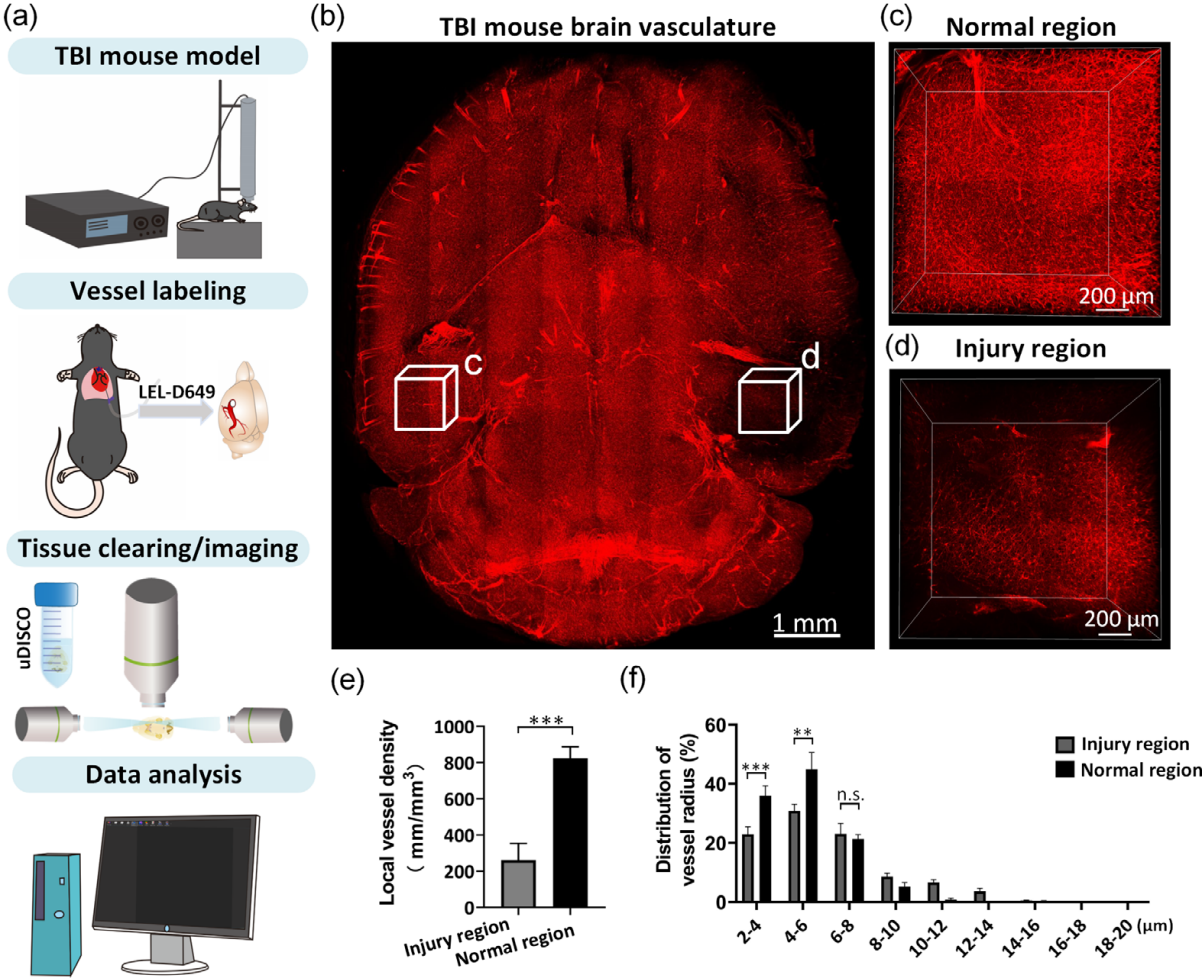
3D visualization and analysis of changes of cerebrovascular structures in TBI mouse brain. (a) Experimental workflow. (b) 3D Reconstruction of the vascular architecture in the brain reveals an obvious vessel information loss in injury area compared with the contralateral normal area. (c) Magnification of region marked in (b). (d) Magnification of region marked in (b). (e) Quantification of vessel densities in normal and injury regions (n=4). (f) Statistical analysis of the distribution of vessel radius in normal and injury regions (n=4). All values are presented as the mean ± SD. Statistical significance in (e) and (f) (***, P<0.001; **, P<0.01) was assessed using an independent-sample t-test.

Figure 6(a) shows the experimental workflow for the construction of the TBI mouse model, vascular labeling, tissue clearing, imaging and data analysis. As expected, the selected vessel labeling/tissue clearing combinations enabled 3D visualization of the vascular networks in TBI mouse brain tissue [Fig. 6(b)]. The 3D reconstruction images revealed substantial loss of vascular structures in the injured region compared with the normal contralateral regions [Figs. 6(c) and 6(d)]. The quantitative data also showed that the vessel length densities decreased obviously in the injured regions [Fig. 6(e)], and the major form of vessel damage occurred in microvessels [Fig. 6(f)]. These results revealed that the selected optimal combinations are also appropriate for studying vasculature under specific pathophysiological conditions.

## 4 Discussions and Conclusions

Given the rapid progress in developing fluorescent labeling strategies and tissue-clearing techniques, the demand for the visualization of detailed vascular structure information in 3D using advanced tissue-clearing and optical imaging has grown. In this study, we systemically evaluated the labeling performances of four commonly used vessel-labeling methods in various organs, as well as their compatibilities with several latest clearing protocols. Based on these quantitative evaluations and comparisons, we selected optimal combinations of vessel labeling and clearing procedures to conduct the 3D reconstruction of vascular networks in typical mouse organs. Our findings should guide researchers in choosing best-fit combinations of labeling and clearing methods to visualize entire vascular networks in different histological and pathological applications.

As mentioned, the four labeling methods selected and evaluated in this study are part of two different labeling strategies: vessel-specific marker vessel labeling and fluorescent dye-filling vessel labeling. Our results reveal that the two different labeling strategies yielded similar brain microvessel labeling patterns; however, signal-to-noise ratios for vessel information acquired through fluorescent dye filling labeling, including Gel-Dex-FITC and DiI, were higher than those of vasculatures labeled using vessel-specific marker injections, such as LEL-D649 and CD31-A647. This discrepancy probably results from the use of more fluorescent solutions during vessel filling (mL level) than during vessel-specific marker injections (μL level), leading to higher concentrations of fluorophores in marked vessels. Additionally, we found that vessel labeling using fluorescent dye perfusions performed better in large vessels than vessel-specific marker injections in typical organs, possibly because the latter simply marks the walls of large vessels with faint signals and leaves the middle of vessels unlabeled. Therefore, vessel labeling using fluorescent dye perfusion is more suited to visualizing both large vessels and capillaries. However, it also should be noted that vessel labeling using fluorescent dye perfusion could cause potential dye leakage, resulting in heterogeneous labeling and missing vessel information.

To achieve 3D mapping of vasculature in different organs, a proper clearing approach that can be applied effectively alongside a given vessel labeling method must be chosen. Therefore, evaluating fluorescence compatibility is essential to selecting the correct combination of vessel labeling and clearing methods. In this study, we chose clearing methods from different strategies: solvent-based methods (FDISCO, uDISCO, and PEGASOS) and aqueous-based methods (CUBIC, PACT, and MACS). The signal-to-background ratios of images, which take both signal and background fluorescence intensities into consideration to express the fluorescence capability of each clearing method, were calculated. In general, fluorescence signals created using injections of lectin and CD31 exhibited the desired compatibility with solvent-based clearing methods, including FDISCO and uDISCO, but they were not well preserved in aqueous-based clearing approaches. Notably, the solvent-based PEGASOS technique also involved aqueous clearing solutions, arguably resulting in unsatisfactory compatibility. DiI solution perfusion-based vessel labeling was only effective with the MACS clearing method due to its excellent lipid preservation ability. By contrast, Gel-Dex-FITC solution perfusion-labeled signals were preserved by all examined clearing methods to different magnitudes, with uDISCO and FDISCO performing better than the other methods.

Based on the results from the comprehensive evaluations, the optimal combination of fluorescent vessel labeling and tissue-clearing methods was selected for the construction of vascular networks in several typical organs. The mouse brain hemisphere labeled using LEL-D649 was cleared employing uDISCO, yielding 3D imaging datasets for the fine reconstruction of brain vascular networks. The vasculature in the mouse liver was labeled through the perfusion of a Gel-Dex-FITC solution due to its robust marking efficiency on liver samples and was cleared using FDISCO. A high-quality 3D map of the liver vasculature was constructed. Similarly, the glomerulus trees in the entire kidney were built utilizing DiI labeling and MACS clearing. Finally, based on the optimal vessel labeling/tissue clearing combinations established, 3D visualization and quantification of structural changes of vascular networks within TBI mouse brains were performed.

In particular, although vessel labeling with transcardial fluorescent dye perfusion marked more large vessels with high signal-to-background ratios than vessel-specific marker injections, this approach can cause specific dye leakage or non-uniform labeling in typical organs. Additionally, the compatibilities of these labeling methods with current clearing techniques are also somewhat unsatisfactory. Recently, novel methods based on fluorescent hydrogels were developed for efficient and robust vascular labeling.^29^^,^^32^ However, the execution of such methods is a bit difficult due to the complexity of hydrogel preparation. Therefore, new more robust, effective, and user-friendly vessel labeling strategies must be developed urgently.

In many studies, transgenic animals expressing endogenous fluorescent proteins (e.g., GFP) are used alongside vascular labeling and tissue clearing. Therefore, comparing the compatibility of endogenous fluorescent proteins with different clearing methods is also valuable. Additionally, optical clearing efficacy is another criterion for choosing a clearing method. These works were not carried out in this study for they have been conducted in our previous publications in which we compare the compatibility of endogenous GFP with different clearing methods on mouse brains and other organs. The clearing efficiencies for different clearing methods on different organs were also quantitatively compared. Therefore, we refer the reader to our previous publications for the comparison data of the compatibility of endogenous GFP with different clearing methods, as well as the clearing efficiencies for different clearing methods on different organs.^33^^,^^34^

This investigation also has several limitations. First, in addition to the two vascular labeling strategies evaluated, another labeling protocol category, i.e., genetically encoded fluorescent protein labeling, was not involved in this study. However, due to potential limitations including the time-consuming nature, high costs, and insufficient compatibility with tissue clearing, we did not include transgenic mice expressing fluorescent proteins in endothelial cells in our quantitative assessment at this stage. Second, intravenous injections are not the only way to deliver LEL and CD31 into blood vessels; passive immersion or immunolabeling employing LEL molecules or CD31 antibodies can also be used to label vessel networks. However, these methods will require a lot of time for diffusion into tissues. Therefore, we did not compare the differences in performance between the two ways using LEL or CD31 antibody labeling. We hope to carry out these tests down the line.

In summary, we assessed the capability of vessel labeling methods from two major categories to label vasculatures in typical mouse organs and tissues, as well as their applicability alongside current tissue-clearing methods. By screening out best-fit vessel labeling and clearing pipelines from multiple aspects for different organs, we obtained fine 3D structural information of various vasculatures. Many studies have used these labeling methods for imaging blood vessels; however, few of them have performed quantitative analysis on vessel density, vessel radius, and vessel length. In this case, our study provides a useful reference for researchers in choosing a proper pipeline for vascular imaging and analysis. This work is expected to provide useful guidance for the histological and pathological investigations of different vasculature and vessel-associated diseases.

## Code, Data, and Materials Availability

The data used in the manuscript are available upon reasonable request to the corresponding author.
